# The Effect of Mindfulness-Based Intervention on Brain-Derived Neurotrophic Factor (BDNF): A Systematic Review and Meta-Analysis of Controlled Trials

**DOI:** 10.3389/fpsyg.2020.02209

**Published:** 2020-09-15

**Authors:** Patama Gomutbutra, Nalinee Yingchankul, Nipon Chattipakorn, Siriporn Chattipakorn, Manit Srisurapanont

**Affiliations:** ^1^Department of Family Medicine, Faculty of Medicine, Chiang Mai University, Chiang Mai, Thailand; ^2^The Northern Neuroscience Center, Faculty of Medicine Chiang Mai University, Chiang Mai, Thailand; ^3^Center of Excellence in Cardiac Electrophysiology Research, Faculty of Medicine, Chiang Mai University, Chiang Mai, Thailand; ^4^Department of Oral Biology and Diagnostic Sciences, Faculty of Dentistry, Chiang Mai University, Chiang Mai, Thailand; ^5^Department of Psychiatry, Faculty of Medicine Chiang Mai University, Chiang Mai, Thailand

**Keywords:** brain-derived neurotrophic factor, BDNF, mindfulness, meditation, neuroplasticity

## Abstract

**Background:** This systematic review aims to answer three questions. First, how much do mindfulness-based interventions (MBIs) affect peripheral brain-derived neurotrophic factor (BDNF)? Second, do mindfulness exercise–based interventions (exercise-MBIs) and mindfulness meditation–based interventions (meditation-MBIs) affect peripheral BDNF differently? Third, does the age of participants and the accumulative hours of MBI practice affect peripheral BDNF?

**Methods:** We included randomized controlled trials comparing MBI and no intervention in adults (age >18 years) who reported peripheral BDNF. Database searches included PubMed, CINAHL, CENTRAL, PsyInfo, and Scopus. Two reviewers independently selected the studies and assessed the trial quality. We used the standardized mean difference (SMD) as the effect size index and conducted moderator analyses.

**Results:** Eleven studies are included in this systematic review. Five studies applying exercise-MBI and three studies applying meditation-MBI are included in the meta-analysis (*N* = 479). The pooled effect size shows a significantly greater increase of peripheral BDNF in MBI groups compared to the control groups (k = 8, N = 479, SMD = 0.72, 95% CI 0.31–1.14, *I*^2^= 78%). Significantly more increases of BDNF in the MBI groups are found in both subgroups of exercise-MBI and meditation-MBI. The effect sizes of both subgroups are not significantly different between subgroups (χ^2^ = 0.02, *p* = 0.88). We find no significant correlation between the effect sizes and the age of participants (*r* = −0.0095, *p* = 0.45) or accumulative hours of MBI practice (*r* = 0.0021, *p* = 0.57).

**Conclusion:** The heterogeneous data of this small sample-size meta-analysis suggests that MBI can increase peripheral BDNF. Either exercise-MBI or meditation-MBI can increase peripheral BDNF.

## Introduction

It is well-established that brain-derived neurotrophic factor (BDNF), a neuronal growth factor, affects neuronal survival and regeneration, called “neuronal plasticity” (Lu et al., 2014). Hence, there is increasing interest in the potential therapeutic effect of interventions that increase BDNF (Bathina and Das, 2015). BDNF is produced in both the central nervous system and peripheral tissues. The BDNF measured from blood, either from serum, plasma, or saliva, is called peripheral BDNF. Previous studies show peripheral BDNF is related to numerous brain disorders. A recent meta-analysis finds that lower levels of peripheral BDNF are associated with an increased risk of depression (Brunoni et al., 2008), Alzheimer's disease (Balietti et al., 2018), Parkinson's disease (Rahmani et al., 2019), and strokes with an unfavorable outcome (Xu et al., 2018). There have been attempts to provide external BDNF, but this has not been successful (Houlton et al., 2019) due to poor blood–brain barrier permeability and short therapeutic half-life (Poduslo and Curran, 1996). Some studies claim that acetylcholinesterase inhibitors and antidepressants may increase peripheral BDNF (Ströhle et al., 2015); however, the evidence still needs to be evaluated (Zhou et al., 2017). Currently, it would be safer and more feasible to promote lifestyle modification that increases BDNF.

Mindfulness, in a focused-attention practice, has been developed to be a health intervention. Mindfulness has been defined as “paying attention in a particular way, on purpose, in the present moment, and non-judgmentally” (Kabat-Zinn, 2003). Mindfulness-based interventions (MBIs) are practices that employ a variety of techniques designed to facilitate mindfulness to affect bodily function and symptoms. Regarding this definition, MBI can be divided into (1) mindfulness-based exercise, which emphasizes body movement, such as yoga, tai chi, and qi gong, and (2) meditation and its derivatives, which emphasize mentality training, such as mindfulness breathing, compassionate body scan, and working with emotions through imagination. This latter type of MBI also includes secular therapy like mindfulness-based stress relaxation (MBSR) and mindfulness-based cognitive therapy (MBCT) (Goldberg et al., 2018).

Numerous studies show that practicing MBI affects brain structure and function. In a meta-analysis of 21 studies, Fox and colleagues applied diffuse tensor imaging and voxel-based morphology imaging MRI to 300 meditation-naïve participants. They find brain changes, namely a moderate increase in brain size, in eight regions. These brain regions are the hippocampus, anterior and midcingulate gyrus, frontopolar cortex, sensory cortices and insula, orbitofrontal cortex, superior longitudinal fasciculus, and corpus callosum (Fox et al., 2014). Another meta-analysis of 18 studies of MBSR and MBCT also finds the improvement of working memory, autobiography memory, and cognitive flexibility after practicing MBI (Lao et al., 2016).

Similar to other interventions affecting brain function, MBIs have been assessed regarding their effects on the brain using the measure of peripheral BDNF. A recent meta-analysis finds that physical exercise, including mindfulness-based exercise (e.g., yoga, tai chi) increased peripheral BDNF (Dinoff et al., 2016). Because mindfulness-based exercise comprises light-to-moderate exercise and mindfulness meditation, it is not yet known if mindfulness meditation plays any role in such an increment (Ainsworth et al., 2000). Another study examining the effect of yoga on peripheral BDNF also reports that the increased peripheral BDNF is negatively correlated with age (r = −0.446) (Pal et al., 2014). In addition, a meta-analysis reports that cumulative hours of physical exercise is an effect modifier of peripheral BNDF increment (Dinoff et al., 2017).

The evidence mentioned above suggests that the effect of MBIs, especially the meditation part, on peripheral BDNF remains inconclusive. This systematic review and meta-analysis aims to answer three questions. First, how much does MBI affect peripheral BDNF? Second, do mindfulness exercise-based interventions (exercise-MBIs) and mindfulness meditation-based interventions (meditation-MBIs) affect peripheral BDNF differently? Third, does the age of MBI practitioners and the accumulative hours of MBI practice affect peripheral BDNF?

## Materials and Methods

The protocol of this systematic review (CRD42018093786) is registered at PROSPERO (International Prospective Register of Systematic Reviews). This present report follows the format of the PRISMA (Preferred Reporting Items for Systematic Reviews and Meta-analyses) Statement.

### Inclusion Criteria of Studies

Studies that are included meet the following criteria: (i) a parallel or crossover randomized, controlled trial (RCT) in adults (age > 18 years); (ii) the experimental group receiving any type of MBI; (iii) the comparator group receiving no treatment, treatment as usual, or being on the waitlist; and (iv) the changes in plasma or serum BDNF after receiving MBI being reported or calculable.

Our meta-analysis does not include non-controlled (single-arm) trials due to the presence of confounding factors. One of the important confounders is called the “vacation effect.” This effect is reported in some studies that show the non-intervention group also had peripheral BDNF changes during follow-up (Epel et al., 2016; Kwak et al., 2019). The BDNF changes suggesting a vacation effect, refers to the temporary improvements in health and psychological well-being after taking a vacation, which soon fade after work resumption (Goldberg et al., 2018). However, improved reliability should result from the balance of confounders in the comparison groups of RCTs. For this reason, it was decided to include only RCTs in our meta-analysis.

### Data Sources and Searches

Searches were conducted on PubMed, EMBASE, The Cochrane Central Register of Controlled Trials (CENTRAL), and CINAHL (nursing and allied health professions), and PsyInfo from inception (2008) up to June 2020. For all full-text articles that passed the screening, their reference lists were examined to identify additional relevant studies (“snowball” method).

### Study Selection and Quality Assessment

Two reviewers (PG and NY) independently selected the searched records, extracted the data, selected the studies, and assessed the trial quality. If any discrepancy existed, other investigators would additionally review and discuss with the first two reviewers to form a consensus.

After the completion of study selection, the data was extracted as recommended by the Center for Reviews and Dissemination. Each trial was evaluated using the Cochrane Collaboration four criteria for assessing the risk of bias. Those include (i) adequate generation of allocation sequence, (ii) concealment of allocation, (iii) prevention of knowledge of the allocated intervention, (iv) dealing with an incomplete data set, (v) selective report, and (vi) other bias.

### Data Extraction

Apart from demographic and clinical characteristics, the changes in peripheral BNDF are the outcome of interest. In comparison to the endpoint peripheral BDNF, the mean changes are preferred because the included studies are likely to have small sample sizes. We declined to use the endpoints of peripheral BDNF because baseline data of paired groups enrolled in a small study might not be comparable although the randomization was applied.

The mean changes and standard deviations (SDs) of peripheral BDNF were extracted. If the study in which the means and SDs of BDNF level changes were not available, the means and SDs at baseline and endpoints were used to calculate the means and SDs of BDNF level changes. To estimate the SD of mean change of BDNF level changes, the baseline and postintervention correlation coefficient (*r*) were needed. If such *r* was not available, *r* was defined as 0.5 for the treatment group and 0.05 for the control group. This estimation was obtained from Ledreux et al. (2019), which is the only study that clearly reports the Pearson *r* for mindfulness practice = 0.439 and Pearson *r* = 0.037 for the control group.

### Meta-Analysis

Because peripheral BDNF can be measured by various laboratory kits and reported in many unit systems, the standardized mean difference (SMD) is used as the effect size index. The present SMD is defined as the difference between the mean change of peripheral BDNF obtained from the experimental group and that obtained from the control groups. This, in turn, is divided by the pooled within-group SD. This is an appropriate index when the subjects are randomly assigned to the comparison groups with the assumption that both groups are equivalent at baseline (Rubio-Aparicio et al., 2018).

**Figure 1:**
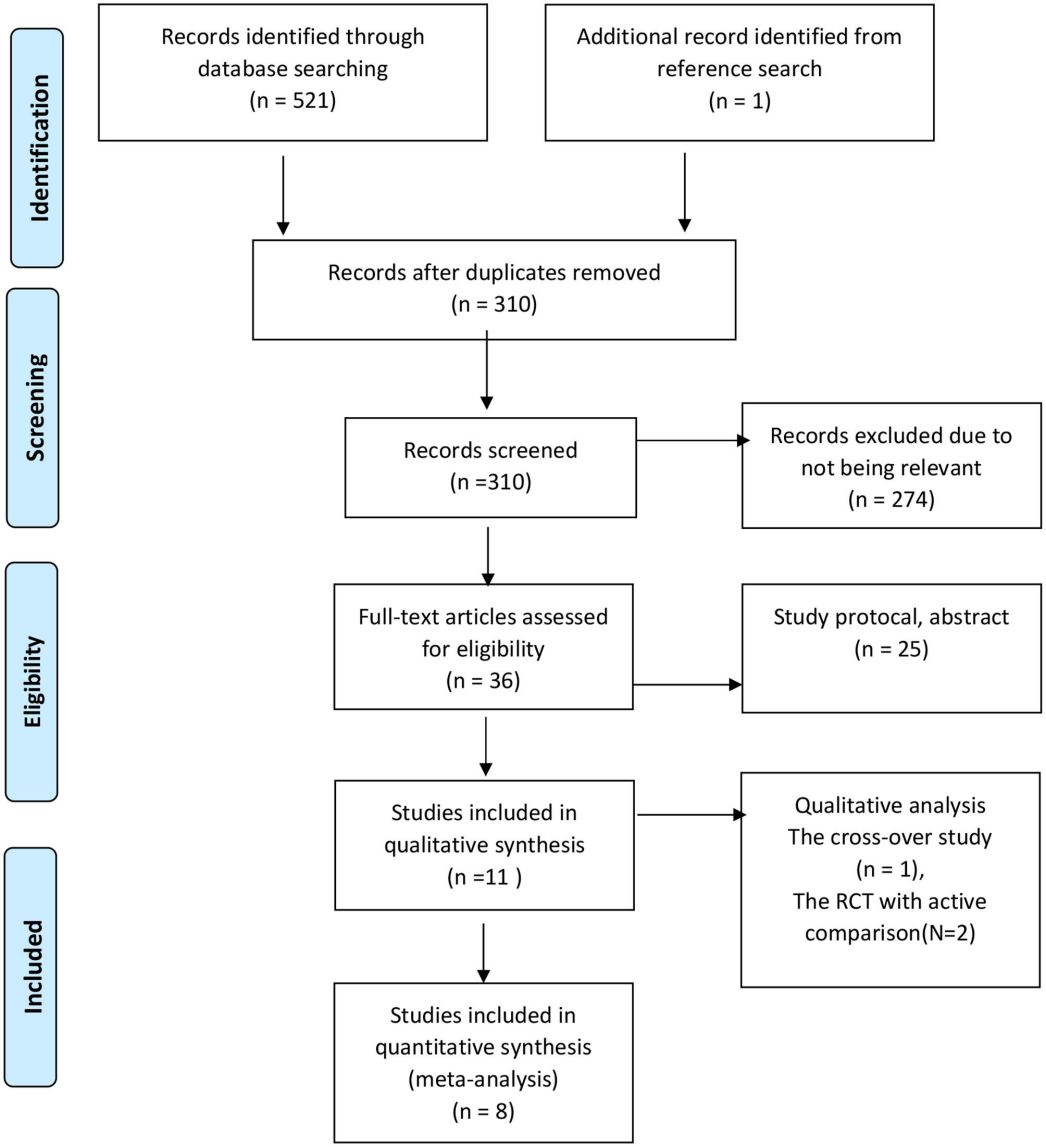
Algorithm of study selection following PRISMA guideline.

Heterogeneity is estimated using the *I*^2^ statistic. If the heterogeneity of data is significantly high (*I*^2^ > 50%), effect sizes are pooled using a random-effect model. The effect sizes are interpreted as Cohen's recommendations: 0–0.19 = negligible effect, 0.20–0.49 = small effect, 0.50–0.79 = moderate effect, 0.80 or more = large effect (Schäfer and Schwarz, 2019). The publication bias is assessed visually using the funnel plot as in Figure 1. The number of trials expected to be able to be included in the meta-analysis is <10. Begg's test for funnel plot symmetrical also applies (Begg and Mazumdar, 1994).

### Moderator Analyses

A subgroup analysis was conducted by separating the included studies into three subgroups based on the types of MBI. These included (i) exercise-MBI (e.g., yoga, tai chi), (ii) meditation-MBI, and (iii) other MBI practices (e.g., MBI added on other programs). Two meta-regression analyses were performed by computing the correlation coefficients (*r*s) between (i) the effect sizes and the average ages of participants and (ii) the effect sizes and the accumulated hours of MBI practice. The accumulated hours of MBI practice of each study were computed by multiplying the practice hours per week with the number of study weeks.

### Software

**Figure 2:**
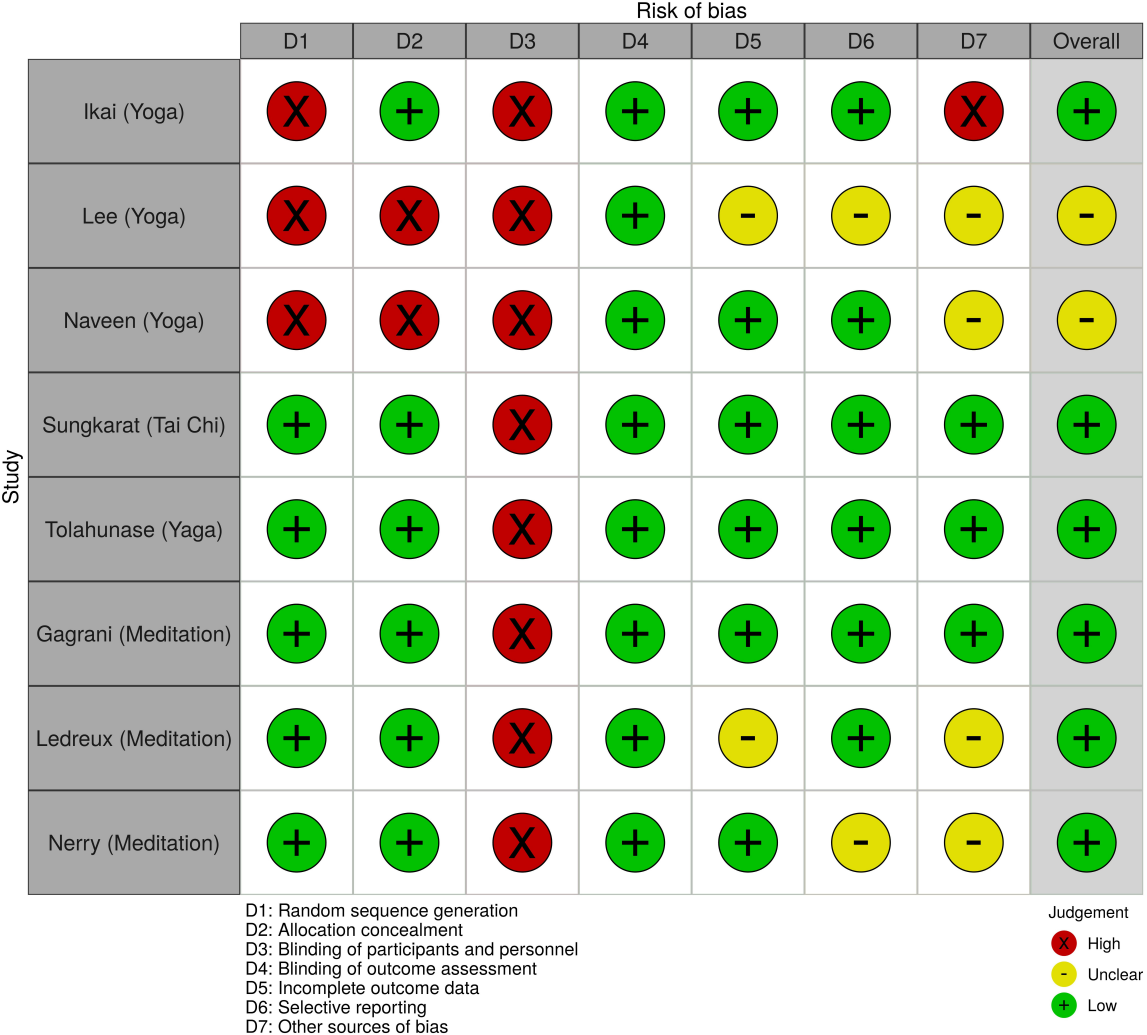
The quality of research.

All analyses and most of Figures were conducted using R 4.0 (R Core Team, 2016) and Figure 2 was produced by Robvis (McGuinness and Higgins, 2020).

## Results

### Study Characteristics and Quality

The searches retrieved 522 records in total (see Figure 1). After the removal of duplicated records, record screening, and assessment of full-text articles, 11 studies met the inclusion criteria and are included in this systematic review. All studies were conducted in adults with a median age of 48 (range 31–84) years, slightly predominantly female with common diagnoses of psychiatric problems and low burdens of physical health. The median accumulative intervention exposure was 28 h (range 8–158 h). Table 1 shows other characteristics of the included studies. The included studies pose a low-to-moderate risk of bias. The common risk is that no study could blind interventions. Figure 2 shows other issues of methodological quality in the included studies.

**Table 1:** The included studies for systematic review (^*^Not included studies in the meta-analysis).

**Study**	**Study design**	**Participant condition and number**	**Average age of participants**	**Intervention**	**Control**	**Accumulative hours of mindfulness intervention practice**	**BDNF baseline and post-intervention**	**BDNF change from baseline comparing intervention and control (effect size, SE)**
**MINDFULNESS BASED EXERCISE**								
Lee et al. (2014)	Parallel RCT	low back pain with depression *N* = 25	40.5 F 100%	Yoga	Regular lifestyle	1 h X 3 times/week X 12 weeks = 36 h	Serum, ng/ml baseline 23.97 post 30.39	1.52 (0.46) Intervention increase more than control
Ikai et al. (2014)	Parallel RCT	Schizophrenia *N* = 50	52.8 F 35%	Yoga + standard treatment	Standard treatment	1 h X 1 times/week × 8 weeks = 8 h	Plasma ng/ml baseline 0.149 post 0.190	−0.06 (0.28) intervention increase less than control
Naveen et al. (2016)	Parallel RCT	Depression *N* = 44	33.5 F 45%	Yoga + standard treatment	standard treatment	1 h X 1 times/week × 12 weeks =12 h	Plasma ng/ml baseline 0.203 post 0.214	0.74 (0.34) Intervention increase more than control
Tolahunase et al. (2018)	Parallel RCT	Depression *N* = 58	39.4 F 15%	Yoga + standard treatment	Standard treatment	120 min × 12 weeks = 168 h	Serum, ng/ml baseline 13.5 post 18.6	1.01 (0.28) Intervention increase more than control
Sungkarat et al. (2018)	Parallel RCT	Mild cognitive impairment *N* = 66	67.5 F 80%	Tai Chi	Regular lifestyle	50 min × 3 times/week × 24 weeks = 60 h	Plasma, ng/ml baseline 0.1123 post 0.385	0.12 (0.24) Intervention increase more than control
**MINDFULNESS MEDITATION AND DERIVATIVES**								
Gagrani et al. (2018)	Parallel RCT	primary angle glaucoma *N* = 60	57.28 F 42%	Meditation	Regular lifestyle	45 min × 7 times/week × 6 weeks = 31.5 h	Serum, ng/ml baseline 52.24 Post 63.25	0.13 (0.25) Intervention increase more than control
Ledreux et al. (Ledreux et al., 2019)	Parallel RCT	Healthy N =78	72.9 F=70%	Meditation	Regular lifestyle	3 h per weeks × 5 week = 15 h	Serum, ng/ml baseline 26.60 post 26.59	1.18 (0.69–1.16) Intervention decrease less than control
Nery et al. (2019)	Parallel RCT	Infertility with anxiety N =62	37.2 F 100%	Meditation	Regular lifestyle	2 h per week for 8 weeks = 16 h	Serum, ng/ml baseline 84.64 post 91.92	0.58 (0.21) Intervention increase more than control
(Håkansson et al., 2016)^*^	Crossover trial control trial	Healthy *N* = 57	70.2 F 58%	Mindfulness practice	Physical exercise, Cognitive training	35 min	Serum, ng/ml Pre-crossing 21.6 Post 21.05	−0.55 (1.27) Mindfulness and cognitive training did not change while physical exercise increase
(Montero-Marin et al., 2019)^*^	Parallel RCT	Fibromyalgia *N* = 24	53.05 F 100%	Attachment-based compassion therapy (ABCT)	Relaxation technique	2 h per week × 8 weeks + 2 h per month × 3 months = 32 h	Serum, ng/ml, baseline 23.03 post 16.03	−1.56 (0.4) Intervention decrease more than control
Siang Ng et al. (2020)^*^	Parallel RCT	Mild cognitive impairment *N* = 55	71.28 F 70%	Mindfulness Awareness Practice (MAP)	Health education Program	1 h per week × 12 weeks + 1 h per month x 6 months =18 h	Serum, log transformation adjusted mean baseline 7.238 post 6.323	−1.61 (0.31) BDNF decrease in both intervention and control group.

* *Not include in met analysis*.

Of 11 included studies, five (Ikai et al., 2014; Naveen et al., 2016, Tolahunase et al., 2017; Sungkarat et al., 2018) and three (Fox et al., 2014; Gagrani et al., 2018; Nery et al., 2019) studies are parallel RCTs applying exercise-MBI and meditation-MBI, respectively. These eight studies had a follow-up duration between 8 and 36 weeks. Due to their similarities, these eight studies are included in the meta-analysis (*N* = 479).

The three following studies (Håkansson et al., 2016; Montero-Marin et al., 2019; Ng et al., 2020) are very dissimilar in character of intervention and control group to the first eight studies. They are included in the qualitative analysis only. Håkansson et al. conducts a crossover study in 19 healthy elders. However, this study was excluded because it applied only active interventions of cognitive training and physical exercise in the control arms. All participants went through different training commercial applications for 35 min each. The study measured the peripheral BDNF within 0, 20, and 60 min after finishing each intervention. They find peripheral BDNF in those receiving physical exercise is significantly increased from a baseline although mindfulness and cognitive training show no significant change. There were two studies related to disease-specific mindfulness programs compared to active interventions. Montero-Martin et al. conducted a parallel RCT in patients with fibromyalgia. They compare attachment-based compassion therapy (ABCT), a fibromyalgia-specific mindfulness-based program with relaxation therapy. The study shows the ABCT group had a significantly greater improved quality of life concurrent with a greater reduction of BDNF. Siang Ng et al. conducted a parallel RCT in elders with mild cognitive impairment. The intervention was a modified MBSR program for the cognitively impaired person called mindful-awareness practice (MAP). The control arm received a lifestyle modification health education program. The study resulted in BDNF in both MAP and control groups reduced from baseline at 3 and 9 months of the program.

### Meta-Analysis

**Figure 3:**
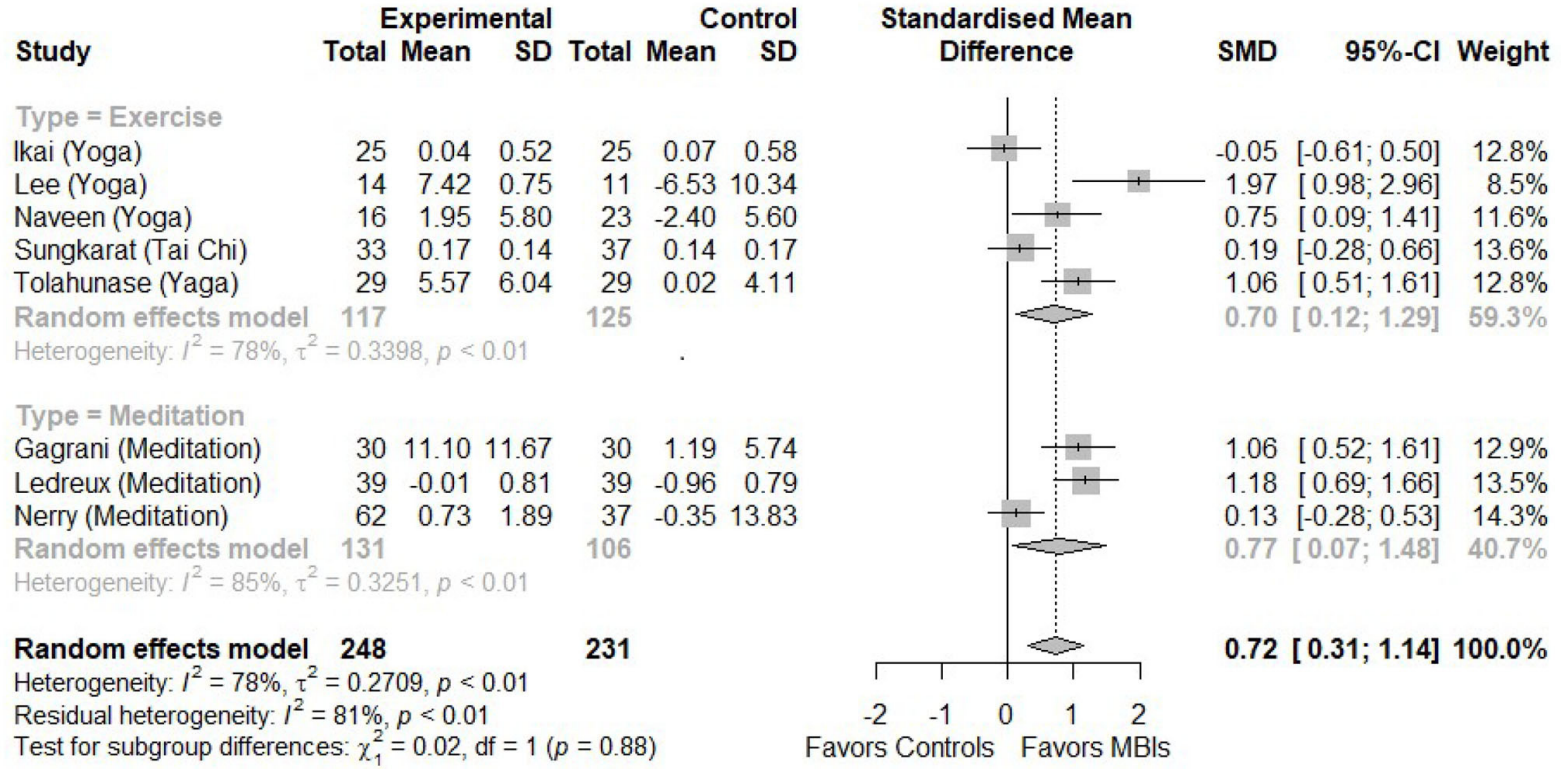
The forest plot of included studies (K = 8).

**Figure 4:**
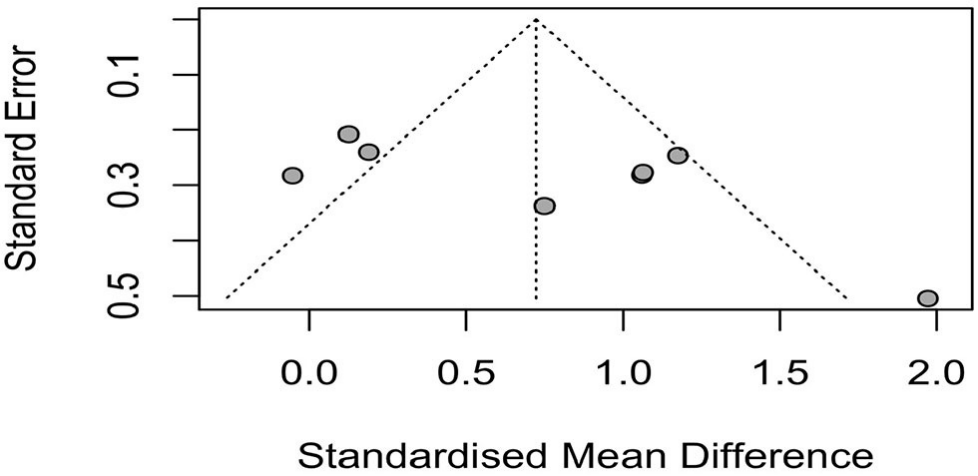
The funnel plot. K = 8, The Egger's test for publication bias: *t* = 1.7856, df = 6, *p* = 0.1244.

Figure 3 shows the meta-analysis of the eight included studies using a random effect model. The pooled effect size shows a significantly greater increase of peripheral BDNF in MBI groups compared to the control groups (k = 8, *N* = 479, SMD = 0.72, 95% CI 0.31–1.14, *I*^2^=78%). Regarding the publication bias, the funnel plot of effect sizes against their standard errors shows a relative symmetry of plots. The Begg's rank test of funnel plot asymmetry indicates no significant asymmetry of effect-size plots (k = 8, z = 0.72, *p* = 0.45) in Figure 4.

### Moderator Analyses

Figure 3 shows the subgroup analysis comparing the pooled effect sizes of BDNF changes between the exercise-MBI and control groups and between the meditation-MBI and control groups. In the MBI groups, significantly greater increases in peripheral BDNF are found in both subgroups (k = 5, N = 242, SMD = 070, 95% CI = 0.12–1.29, *I*^2^ = 78%) and meditation-MBI (k = 3, N = 237, SMD = 077, 95% CI = 0.07–1.48, *I*^2^ = 85%). The effect sizes of both subgroups are not significantly different between subgroups (χ^2^ = 0.02, *p* = 0.88).

**Figure 5:**
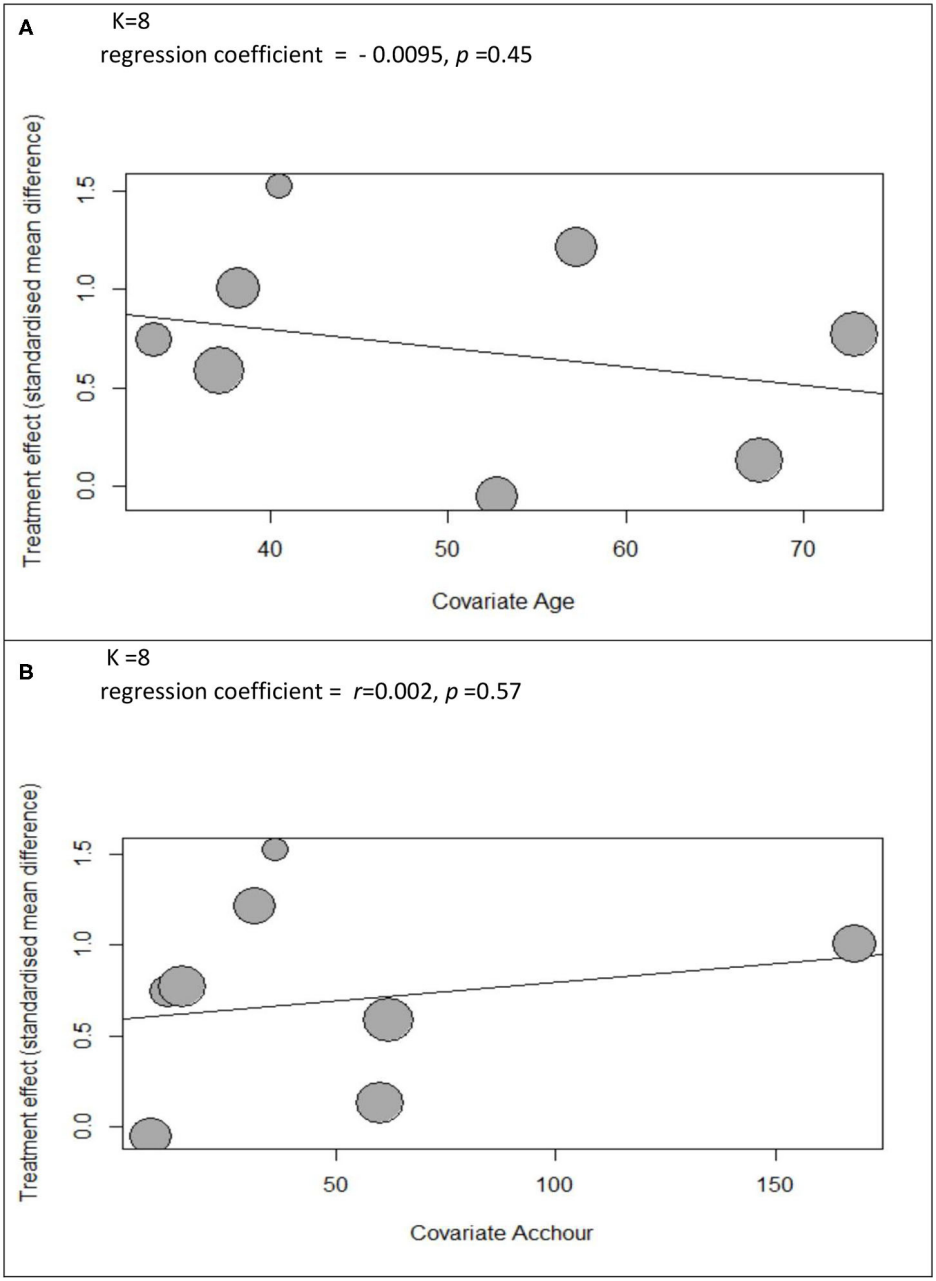
The bubble plots of met regression analysis of **(A)** participants' age and **(B)** accumulated hours of practice.

The meta-regression analyses shows no significant correlation between the effect sizes and participants' age (*r* = −0.0095, *p* = 0.45) or accumulative hours of practice (*r* = 0.0021, *p* = 0.57) (see the bubble plot in Figure 5).

## Discussion

Our meta-analysis examines the effect of MBI on peripheral BDNF by including eight RCTs (*N* = 479). Based on the heterogeneous data, it is found that MBIs can increase peripheral BDNF. The increases in peripheral BDNF are not different between those practicing exercise-MBI and meditation-MBI.

The practitioners' age or the accumulative hours of MBI practice do not appear to be effect modifiers on peripheral BDNF affected by MBIs.

The present evidence supports previous findings and extends our knowledge in this area. The present meta-analytic results are in line with the report that mild-to-moderate exercise incorporated in MBI (e.g., yoga, tai chi) can increase peripheral BDNF. However, our subgroup analytic results extend current knowledge that not only exercise-MBI but also meditation-MBI can increase BDNF. This new knowledge suggests that, for exercise-MBI, both components of exercise and meditation contribute to the increase of peripheral BDNF.

Our meta-regression analytic results suggest that effect modifiers of peripheral BDNF changes affected by physical exercise may not be applicable for MBI. Previous studies report that practitioners' age and hours of practice are associated with the changes in peripheral BDNF related to yoga or exercise practice (Dinoff et al., 2016; Lao et al., 2016). However, our meta-analyses does not show the association of those effect modifiers in MBI practitioners. It is possible that the inclusion of three meditation studies, which are non-exercise-based interventions, may reduce the strength of such association. In addition, the small sample sizes of the included studies might cause a type II error, which results in the discovery of false-negative findings.

Håkansson et al. (2016) shows that a short bout of mindfulness does not change peripheral BDNF. He hypothesizes that physical exercise and mindfulness meditation may increase blood BDNF via a different mechanism. This may imply the relatively delayed effect of mindfulness on peripheral BDNF. One possibility is that blood BDNF increases from BDNF efflux from the brain. However, this hypothesis has a counter-argument; because BDNF has a relatively short half-life (around 45 min), it may disappear before getting through the blood–brain barrier (Poduslo and Curran, 1996; Pan et al., 1998). Another possible explanation is that mindfulness can reduce systemic inflammation and free radicals, which help reduce BDNF eradication (Yang et al., 2017). This is supported by a previous meta-analysis, in which MBI, both exercise-MBI and meditation-MBI, show a significant correlation with clinical improvement. Concurrently, there is also a significant correlation with reduced inflammatory biomarkers, specifically salivary levels of interleukin 6 and tumor necrosis factor-alpha in depression and generalized anxiety disorder (Hofmann et al., 2010; Sanada et al., 2020).

There might be a unique interaction between MBI and chronic pain. A study in patients with fibromyalgia (Montero-Marin et al., 2019) shows participants had decreased BDNF concurrent with improving pain symptoms after the intervention and active intervention control. The author refers to a result supported by clinical observations that, in patients with central sensitivity pain syndrome, during the active symptoms, peripheral BDNF is higher than when symptoms are improving (Deitos et al., 2015). Preclinical studies find that BDNF might be involved in maladaptive mechanisms in neuropathic pain, spasticity, and convulsive activity (Smith, 2014). Hence, more studies on the effect of MBI on BDNF in chronic pain syndrome are warranted.

The low adherence to mindfulness practice can mask the effectiveness of MBI for BDNF. Ng et al. report decreased BDNF in both intervention and control groups after 9-months follow-up. The author discusses that low compliance to homework mindfulness practice in mild cognitive impairment might influence the result. This is supported by a meta-analysis that finds adherence to home practice significantly impacts the effectiveness of MBI (Parsons et al., 2017). This lesson highlights the need to explore the optimum dose response for mindfulness practice to balance between achieving desired outcomes and being suitable for the participant's feasibility.

There are some limitations to this current systematic review. First, our review includes a smaller number of RCTs and participants compared to previous systematic reviews that include all trials examining the changes of peripheral BDNF affected by some interventions, e.g., physical exercise (Dinoff et al., 2016). This limitation may cause some type II errors in our statistical analyses, e.g., meta-regression analyses. As mentioned in the present method section, for the studies in this area, the data as well as the meta-analyses obtained from RCTs would be more reliable. However, second, the majority of participants in our meta-analysis are mentally ill and have a low burden of physical health problems. The generalizability of the present results to other groups of patients may be limited. Third, there is a lack of detailed information about factors that may affect peripheral BDNF across each study discussed before; such as the intervals between the last session of intervention and blood obtainment, the proportion of participants with chronic pain syndrome, and adherence to the mindfulness program. Last, the exclusion of non-English articles might raise the risk of publication bias.

More studies in this area remain needed. These include head-to-head RCTs comparing the effects of exercise-MBI and meditation-MBI on peripheral BDNF. Because BDNF activities are also related to physical illnesses, studies of metabolic syndrome as well as patients with chronic pain syndrome should be also carried out.

Despite the above limitations, the present findings are still helpful for clinical practice, in particular, the patients with a physical disability. Increased BNDF may decrease emotional problems. Meanwhile, more exploration is needed on the influence of chronic pain syndrome and the aging brain on the effect of MBI on BDNF. Many people may wish to increase their BDNF. For an individual without a physical disability, he/she can practice physical exercise to increase his/her BDNF. However, those who have a physical disability may choose to practice meditation-MBI to increase their BDNF.

## Conclusions

The heterogeneous data of this small sample size meta-analysis suggest that MBI can increase peripheral BDNF. Either mindfulness exercise or mindfulness meditation-based intervention can increase peripheral BDNF. Patients with a physical disability may choose to practice MBI to increase their BDNF. More studies in this area are warranted.

## Data Availability Statement

The original contributions presented in the study are included in the article/Supplementary files, further inquiries can be directed to the corresponding author.

## Ethics Statement

This study meets the criteria of Chiang Mai University for ethical approval and consent exemption (Exemption number 2561-05447).

## Author Contributions

PG and MS designed the study. PG and NY conducted the research. PG and MS conducted the meta-analysis. PG wrote the first draft of the manuscript. NC, SC, and MS participated in the revision of the subsequent draft. All authors read and approved the final manuscript.

## Conflict of Interest

The authors declare that the research was conducted in the absence of any commercial or financial relationships that could be construed as a potential conflict of interest.
