# Supplementary material for: The Effect of Mindfulness-Based Intervention on Brain-Derived Neurotrophic Factor (BDNF): A Systematic Review and Meta-Analysis of Controlled Trials
Source: Front Psychol. 2020 Sep 15;11:2209. doi: 10.3389/fpsyg.2020.02209 (PMC7522212; doi:10.3389/fpsyg.2020.02209)
Supplement: Supplementary file 1 [file Presentation_1.pdf]

Supplementary : Searching term

1. MeSH

("mindfulness"[MeSH Terms] OR "mindfulness"[All Fields]) OR ("meditation"[MeSH Terms] OR "meditation"[All Fields])) OR "Mind-Body Therapies"[Mesh]) OR "Spiritual Therapies"[Mesh]) OR ("yoga"[MeSH Terms] OR "yoga"[All Fields])) OR ("tai ji"[MeSH Terms] OR ("tai"[All Fields] AND "ji"[All Fields]) OR "tai ji"[All Fields] OR ("tai"[All Fields] AND "chi"[All Fields]) OR "tai chi"[All Fields])) OR "Exercise Movement Techniques"[Mesh]) OR (("mindfulness"[MeSH Terms] OR "mindfulness"[All Fields]) AND based[All Fields] AND ("Stress"[Journal] OR "stress"[All Fields]) AND reduction[All Fields])) OR MBSR[All Fields]) AND (("brain-derived neurotrophic factor"[MeSH Terms] OR ("brain-derived"[All Fields] AND "neurotrophic"[All Fields] AND "factor"[All Fields]) OR "brain-derived neurotrophic factor"[All Fields] OR ("brain"[All Fields] AND "derived"[All Fields] AND "neurotrophic"[All Fields] AND "factor"[All Fields]) OR "brain derived neurotrophic factor"[All Fields]) OR ("brain-derived neurotrophic factor"[MeSH Terms] OR ("brain-derived"[All Fields] AND "neurotrophic"[All Fields] AND "factor"[All Fields]) OR "brain-derived neurotrophic factor"[All Fields] OR "bdnf"[All Fields]))) AND ((((((("Stress"[Journal] OR "stress"[All Fields]) OR "Stress, Psychological"[Mesh]) OR "Depression"[Mesh]) OR ("Cogn Int Conf Adv Cogn Technol Appl"[Journal] OR "cognitive"[All Fields])) OR "Cognition"[Mesh]) OR "Sleep"[Mesh]) OR "pain"[Mesh]) OR "Anxiety"[Mesh])

2. CINAHL

S1 mindfulness OR Meditation OR Mind Body Therapy OR Spiritual Therapy OR Yoga OR tai chi OR mindfulness based stress reduction OR mbsr OR hypnosis OR acupuncture OR massage OR Qi gong OR Art Therapy OR Music Therapy OR Cognitive Therapy
S2 brain derived neurotrophic factor OR nerve growth factor OR bdnf
#s1 AND #s2

3. Cochran library

#1 mindfulness OR Meditation OR Mind Body Therapy OR Spiritual Therapy OR Yoga OR tai chi OR mindfulness based stress reduction OR mbsr OR hypnosis OR acupuncture OR massage OR Qi gong OR Art Therapy OR Music Therapy OR Cognitive Therapy

#2 Brain-Derived Neurotrophic Factor OR BDNF OR Nerve Growth Factor NOT gene #3 #2 AND #1
